# Overexpression of MYB drives proliferation of CYLD‐defective cylindroma cells†

**DOI:** 10.1002/path.4717

**Published:** 2016-04-21

**Authors:** Neil Rajan, Mattias K Andersson, Naomi Sinclair, André Fehr, Kirsty Hodgson, Christopher J Lord, Dmitry V Kazakov, Tomas Vanecek, Alan Ashworth, Göran Stenman

**Affiliations:** ^1^ Institute of Genetic MedicineNewcastle UniversityNewcastle upon TyneUK; ^2^ Sahlgrenska Cancer Centre, Department of PathologyUniversity of GothenburgSweden; ^3^ Breakthrough Breast Cancer Research CentreInstitute of Cancer ResearchLondonUK; ^4^ Sikl's Department of PathologyCharles University in Prague, Medical Faculty in PilsenCzech Republic; ^5^ UCSF Helen Diller Family Comprehensive Cancer CenterSan FranciscoCAUSA

**Keywords:** cylindroma, adenoid cystic carcinoma, germline mutation, CYLD, MYB, MYB–NFIB, gene fusion

## Abstract

Cutaneous cylindroma is an adnexal tumour with apocrine differentiation. A predisposition to multiple cylindromas is seen in patients with Brooke–Spiegler syndrome, who carry germline mutations in the tumour suppressor gene CYLD. Previous studies of inherited cylindromas have highlighted the frequent presence of bi‐allelic truncating CYLD mutations as a recurrent driver mutation. We have previously shown that sporadic cylindromas express either MYB–NFIB fusion transcripts or show evidence of MYB activation in the absence of such fusions. Here, we investigated inherited cylindromas from several families with germline CYLD mutations for the presence of MYB activation. Strikingly, none of the inherited CYLD‐defective (n = 23) tumours expressed MYB–NFIB fusion transcripts. However, MYB expression was increased in the majority of tumours (69%) and global gene expression analysis revealed that well‐established MYB target genes were up‐regulated in CYLD‐defective tumours. Moreover, knock‐down of MYB expression caused a significant reduction in cylindroma cell proliferation, suggesting that MYB is also a key player and oncogenic driver in inherited cylindromas. Taken together, our findings suggest molecular heterogeneity in the pathogenesis of sporadic and inherited cutaneous cylindromas, with convergence on MYB activation. © 2016 The Authors. *The Journal of Pathology* published by John Wiley & Sons Ltd on behalf of Pathological Society of Great Britain and Ireland.

## Introduction

Recurrent gene fusions linking the oncogene *MYB* and the transcription factor gene *NFIB* have been described in the vast majority of adenoid cystic carcinomas (ACCs) of the breast and head and neck 1, 2, 3. The MYB–NFIB oncoproteins retain the DNA‐binding and transactivation domains of MYB and likely drive an oncogenic transcription programme 1. The wild‐type MYB protein has an important role as a regulator of stem/progenitor cells in the bone marrow and in colonic crypts, and is overexpressed in several neoplasms, including colon and breast carcinomas and subsets of T cell acute lymphoblastic leukaemia 4.

Sporadic dermal cylindromas are cutaneous adnexal tumours that occasionally share histological features with ACC. Sporadic cylindromas express *MYB–NFIB* fusion transcripts and protein at a lower frequency, and occasionally demonstrate *CYLD* mutations 5, 6. Multiple cylindromas are seen in patients with Brooke–Spiegler syndrome (BSS), who carry germline mutations in the tumour suppressor gene *CYLD*. These patients develop cylindromas from puberty onwards, typically on the head and neck. Interestingly, these tumours show a striking architectural arrangement of tumour cells, namely, a cylindrical pattern similar to that seen in ACC. Spiradenomas that are both clinically and histologically distinct in appearance have been shown to grow in contiguity with cylindromas in BSS patients, and hybrid lesions, called spiradenocylindromas, are also a common feature 7. Recent molecular characterization of these tumours highlighted the relative paucity of genetic changes compared to other solid tumours. These were limited to germline mutations in *CYLD* and loss of heterozygosity at the *CYLD* locus 7. Notably, < 5% of patients with germline *CYLD* mutations who develop multiple cylindromas also develop membranous basal cell adenomas (MBCA) of the salivary glands 8. MBCA, which accounts for 1–2% of all salivary gland tumours in the general population, has striking histological similarities with cylindroma 8.

We chose to explore the presence of *MYB–NFIB* fusion transcripts in tumours from patients with germline *CYLD* mutations, to determine whether such fusions were present at a similar frequency to that seen in sporadic cylindroma and ACC, which share histological similarities. We found that inherited cylindromas do not harbour *MYB–NFIB* gene fusions but instead show frequent overexpression of *MYB* mRNA and protein. Notably, we also show that MYB drives the proliferation of *CYLD*‐defective cylindroma cells. Our findings highlight that different tumourigenic events may ultimately converge on MYB, and that this oncoprotein represents a putative therapeutic target in sporadic and inherited cylindromas as well as in ACC.

## Materials and methods

### Tumour samples

Fresh‐frozen (cases 1–13) and formalin‐fixed, paraffin‐embedded (FFPE; cases 14–23) tumour tissues were obtained from patients undergoing the surgery indicated to control tumour burden or for symptomatic relief, under regional ethical committee approval (Newcastle upon Tyne, UK, Rec. Ref. 06/1001/59; and Pilsen, Czech Republic, EC Approval date 13 December 2007). Pertinent clinicopathological information and *CYLD* mutation status of the 23 patient samples are shown in Table 1; 10 patients and/or their samples have been a subject of four previous studies 9, 10, 11, 12.

**Table 1:** Clinicopathological and MYB–NFIB fusion data on 23 CYLD‐defective tumours from 15 patients

Case no.	Germline *CYLD* mutation	Sex/age	Diagnosis	Tumour material	*MYB–NFIB* fusion transcript
**1** #	c.2460delC	F/78	Cylindroma	FF	Negative
**2**	c.2806C > T	F/63	Cylindroma	FF	Negative
**3** ##	c.2460delC	F/42	Cylindroma	FF	Negative
**4** #	c.2460delC	F/78	Cylindroma	FF	Negative
**5**	c.2460delC	F/58	Cylindroma	FF	Negative
**6** #	c.2460delC	F/78	Cylindroma	FF	Negative
**7** #	c.2460delC	F/78	Cylindroma	FF	Negative
**8** #	c.2460delC	F/78	Cylindroma	FF	Negative
**9** #	c.2460delC	F/78	Cylindroma	FF	Negative
**10** ##	c.2460delC	F/42	Cylindroma	FF	Negative
**11**	c.2469 + 1 G > A	F/85	Cylindroma	FF	Negative
**12** ##	c.2460delC	F/42	Cylindroma	FF	Negative
**13** ##	c.2460delC	F/42	Cylindroma	FF	Negative
**14**	Unknown (Patient 55 in Grossmann *et al.* 9)	F/59	Cylindroma	FFPE	Negative*
**15**	c.2108G > C (Patient 21 in Grossmann *et al.* 9)	M/34	Cylindroma	FFPE	Negative*
**16**	c.2806C > T (Patient 48 in Grossmann *et al.* 9)	F/44	Cylindroma	FFPE	Negative*
**17**	c.2041 + 1 G > T (Kacerovska *et al.* 10)	F/64	Cylindroma	FFPE	Negative*
**18**	Unknown**	F53	Spiradeno‐cylindroma	FFPE	Negative*
**19**	Large 13.6 kb deletion (Patient 67 in Grossmann *et al.* 9)	F/47	Spiradeno‐cylindroma	FFPE	Negative*
**20**	c.2108G > C (Patient 22 in Grossmann *et al.* 9)	F/59	Spiradeno‐cylindroma	FFPE	Negative*
**21**	Not performed (Sister of patient 19)	F/33	Spiradenoma	FFPE	Negative*
**22**	Not performed (Daughter of patient 20 in Kazakov *et al.* 11 who was demonstrated to carry a heterozygous change in *CYLD*; c.2806C > T)	F/51	Spiradenoma	FFPE	Negative*
**23**	c.1961 T > A (Patient 18 in Grossmann *et al.* 9)	M/57	Spiradenoma	FFPE	Negative*

All tumours were negative for *MYB–NFIB* fusion transcripts by RT–PCR screening.

# Six tumours from the same patient.

## Four tumours from the same patient.

* No MYB rearrangements by FISH.

** Not done, but the patient had multiple tumours on the scalp.

FF, Fresh‐frozen tumour tissue; FFPE, formalin‐fixed. paraffin‐embedded tumour tissue.

### RT–PCR and nucleotide sequence analysis

Total RNA was extracted from five 10 µm sections obtained from fresh‐frozen tumour specimens (cases 1–13), using the RNeasy Mini‐kit (Qiagen, Hilden, Germany) and from five 10 µm FFPE sections from cases 14–23. The FFPE sections were deparaffinized and RNA was isolated using the RNeasy FFPE Kit (Qiagen). All RNAs were subsequently converted to cDNA using the SuperScript^®^ First‐Strand Synthesis System (Invitrogen, Karlsruhe, Germany) with random hexamer primers, as recommended by the manufacturer. As a control for intact RNA and cDNA, RT–PCR reactions for expression of *UBC* (amplification of a 150 bp product), *18S* (amplification of a 187 bp product) or *GAPDH* (amplification of a 290 bp product) were performed on all cDNAs.

All tumours were screened for the most common *MYB–NFIB* fusion transcript variants, ie *MYB* exon 14 fused to *NFIB* exons 8a, 8c or 9, respectively, and *MYB* exon 12 fused to *NFIB* exon 9. The *MYB* and *NFIB* exons were numbered as described elsewhere 1. All PCR primers used are shown in Table S1 (see supplementary material) or have been described previously 1. As a positive control, two ACCs with known *MYB–NFIB* fusion transcripts were used 1. Each *MYB–NFIB* RT–PCR was repeated in triplicate.

### Fluorescence in situ hybridization (FISH) analysis

FISH analysis for detection of *MYB* gene rearrangements was performed on 5 µm FFPE sections, using a dual‐colour *MYB* split FISH probe (Abnova, Taipei City, Taiwan). The protocols for pretreatment, hybridization and post‐hybridization washes were essentially as recommended by the manufacturer. Fluorescence signals were digitized, processed and analysed using the Isis FISH imaging system v. 5.5 (MetaSystems GmbH, Altlussheim, Germany). At least 50 nuclei were scored from each case.

### Microarray data

Genome‐wide transcriptomic profiles of 32 *CYLD*‐defective tumours were generated, using RNA extracted from fresh‐frozen microdissected tissues, as previously described 7. In brief, a bead microarray platform was used to obtain the gene expression values from 32 tumour samples and 10 perilesional controls. Control tissue consisted of epidermis, hair follicles and eccrine/apocrine glands. The Illumina WG‐DASL platform (San Diego, CA, USA; http://www.illumina.com) allowed for monitoring of the gene expression of 24 526 transcripts; 50 ng total RNA from each sample was converted to cDNA according to the supplied protocol and then hybridized to Illumina Human 8v3 chips. A confocal Illumina Infinium bead reader was used to scan the chips and the signal values were extracted using Illumina Beadstudio software v. 3.0. Microarray data associated with this study are accessible at: http://www.ebi.ac.uk (Accession No. E‐MTAB‐352).

### Data normalization and analysis

Raw gene expression values were normalized using quantile normalization. Differential expression analysis was performed using Beadstudio software to detect significant differentially expressed genes. Differences in the signal values for each gene were scored with a differential score (DS). Bonferroni testing was performed to correct for multiple hypothesis testing, and the results were ranked in order of differential score, with a differential score in the range +374 to −374, with ± 65 corresponding to a cut‐off of *p* < 0.01 and ± 13 corresponding to *p <* 0.05.

### Immunohistochemistry

FFPE tissue sections were dewaxed with xylene and rehydrated in a graded ethanol series before undergoing microwave antigen retrieval in sodium citrate buffer, pH 6.0. Tissue sections were blocked with peroxidase blocking agent (Dako, Cambridgeshire, UK) and then incubated with an anti‐MYB antibody, ab17851 (Abcam, SPM 175). Tissue sections were thereafter washed in phosphate‐buffered saline (PBS) and probed with secondary HRP‐conjugated antibodies, and staining was visualized with 3,3′‐diaminobenzidine (DAB). Haematoxylin was used as a nuclear counterstain. The samples were assessed for MYB positivity; if > 15% of cells demonstrated increased expression of MYB, they were considered positive.

### Protein expression quantification

Five representative immunohistochemistry images were taken from each tumour sample and control areas using a Zeiss Axioimager (Zeiss, UK). ImageJ software (v. 1.48 g; http://imagej.nih.gov/ij) was used to quantify DAB staining. RGB values that were restricted to the colour of DAB in our staining protocol were used to select for areas in images that were DAB‐positive. The area of the positive image was then quantified and compared to the total area assessed to generate a ratio of positive staining. The mean of the ratios was used to generate a ratio per sample. The values in the tumours were then compared with the values in the controls, using a *t*‐test.

### Cylindroma cell culture and siRNA transfection

Primary *CYLD*‐defective cylindroma cells were derived from BSS patients as previously described 7, and were propagated in keratinocyte–SFM medium supplemented with penicillin (100 U/ml) and streptomycin (100 µg/ml) (Thermo Fischer Scientific) at 37 °C and 5% CO_2_ in 12.5 cm^2^ flasks (BD Falcon), or in assay plates (see below) coated using a Coating Matrix Kit (Thermo Fischer Scientific). Cylindroma cells were subsequently transfected with 50 nm Stealth *MYB* siRNAs (HSS106819 and HSS106821) or negative control siRNAs, using the Lipofectamine RNAiMAX transfection reagent (all from Thermo Fisher Scientific) in antibiotic‐free medium, according to the manufacturer's instructions.

### Reverse‐transcription quantitative RT–PCR (RT–qPCR)

*CYLD*‐defective cylindroma cells were treated with *MYB* siRNAs for 48 h before total RNA was extracted using an RNeasy Micro‐kit (Qiagen). RNA was reverse‐transcribed using an iScript cDNA Synthesis Kit (Bio‐Rad Laboratories) and quantitative real‐time PCR (qPCR) analysis was performed using the AB 7500 Fast Real‐time PCR System (Thermo Fisher Scientific) with TaqMan Gene Expression Assays (Thermo Fisher Scientific) for *MYB* (Hs00920554_m1), *BCL2* (Hs00608023_m1) and *BIRC3* (Hs00154109_m1), and *GUSB* (Hs99999908_m1) as a reference gene. The relative expression levels of the genes were calculated using the ΔΔ*C* t method 13.

### Western blotting

*CYLD*‐defective cylindroma cells were treated with *MYB* siRNAs for 72 h, after which total cellular protein was prepared by lysing cells in RIPA lysis buffer (Millipore) supplemented with complete Mini Protease Inhibitors (Roche). Protein concentrations in lysates were determined using a DC Protein Assay Kit (Bio‐Rad Laboratories). Protein samples were diluted for equal loading and analysed by western blotting, using the NuPage system (Life Technologies) on 4–12% Bis‐Tris gels. Proteins were blotted onto PVDF membranes and probed with mouse monoclonal antibodies for MYB (1–1, Merck Millipore) and β‐actin (ab8227, Abcam). Bands were visualized using horseradish peroxidase‐conjugated secondary antibodies by chemiluminescent detection with a SuperSignal West Femto Maximum Sensitivity Substrate (Thermo Fisher Scientific), and subsequently imaged using a LAS‐4000 imaging system (Fujifilm).

### Cell proliferation assay

*CYLD*‐defective cylindroma cells were treated with *MYB* siRNAs for 6 consecutive days, after which cell proliferation was assayed using Alamar Blue Reagent (Thermo Fisher Scientific) and a VICTOR‐3 multilabel reader (Perkin‐Elmer), according to instructions supplied by the manufacturers.

## Results

### CYLD‐defective tumours do not express MYB–NFIB fusion transcripts

To determine whether inherited, *CYLD*‐defective tumours express *MYB–NFIB* fusion transcripts, we adopted a validated RT–PCR strategy for exploring the presence of such transcripts in a collection of 13 snap‐frozen and 10 FFPE tumours derived from patients with BSS with confirmed *CYLD* mutations. The mean age of the patients was 55.9 years and the tumours (*n =* 23) were from 15 patients (13 females and two males) from 11 genetically‐defined families (Table 1). None of the tumour samples expressed any of the *MYB–NFIB* fusion transcript variants tested for (data not shown). In contrast, the positive controls from fusion‐positive ACCs expressed the expected *MYB–NFIB* fusion transcript variants (data not shown).

**Figure 1:**
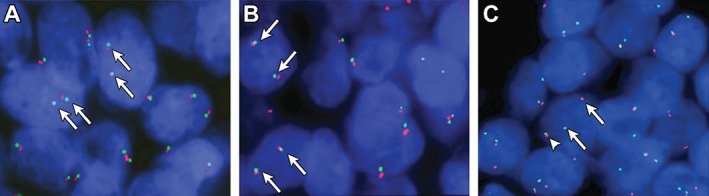
**(**A, B) FISH analysis of two inherited cylindromas, showing two non‐rearranged copies of MYB in each cell nucleus (fused red/green signals indicated by arrows). (C) FISH analysis of a MYB–NFIB fusion‐positive adenoid cystic carcinoma (control) with a split MYB signal consistent with a MYB gene fusion; arrows, separated red and green signals; arrowhead, intact MYB allele

To identify possible rearrangements of the *MYB* locus, we performed FISH analysis of FFPE sections from 10 *CYLD*‐defective tumours (cases 14–23). Using a dual‐colour *MYB* break‐apart probe, we did not detect any evidence of rearrangements or copy number gains/amplifications involving the *MYB* locus (Figure 1).

### MYB protein expression is up‐regulated in inherited CYLD‐defective tumours

**Figure 2:**
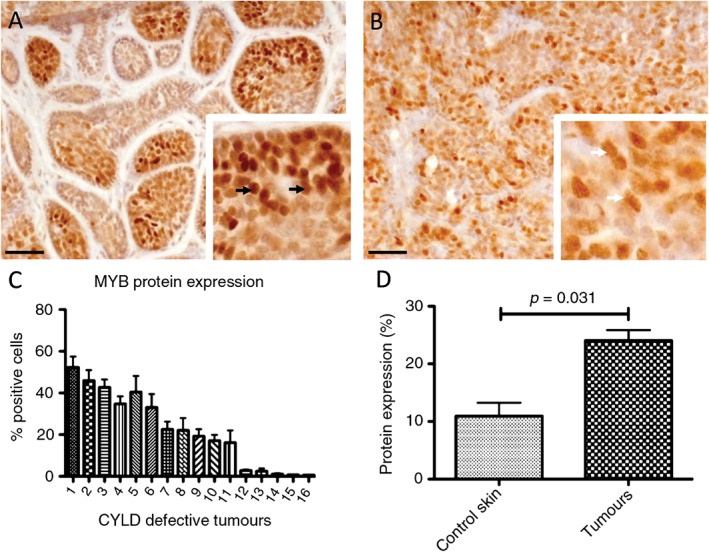
MYB protein expression in inherited CYLD‐defective tumours. FFPE tissue sections of cylindromas and spiradenomas (n = 16) and perilesional control skin were immunostained with an anti‐MYB antibody. (A, B) MYB protein expression in (A) cylindromas and (B) spiradenomas, demonstrating nuclear localization (black arrows in inset) and increased intensity of staining when compared to perilesional tissue; scale bars = 50 µm. (C) Of the cases, 69% (11/16) were positive for MYB. (D) Protein expression of MYB was significantly increased in cylindromas and spiradenomas compared to controls

We assessed MYB protein expression in inherited CYLD‐defective tumours (*n =* 16) to determine the frequency of MYB‐positive tumours in this group. These tumours included cylindromas and spiradenomas from patients with germline mutations in *CYLD*. Tissue sections were probed with anti‐MYB antibody and protein expression was visualized using immunohistochemistry (Figure 2A, B). Eleven of 16 tumour samples (69%) were MYB‐positive, ie they showed > 15% positive cells (Figure 2C). MYB protein expression in cylindromas (Figure 2A) and spiradenomas (Figure 2B) demonstrated nuclear localization (white arrows in inset) and increased intensity of staining when compared to perilesional tissues (see supplementary material, Figures S1A, S2). Immunopositive cylindroma cells were seen in parts of each island, and were not restricted to the basal cells. In MYB‐positive spiradenomas, the expression was also variable in intensity, with positive cells scattered throughout the specimens (see supplementary material, Figure S1B). Similar MYB staining patterns were seen in *MYB–NFIB* fusion‐positive and ‐negative sporadic cylindromas (see supplementary material, Figure S1C, D). Semi‐quantitative assessment of protein expression by measurement of immunostaining intensity, using ImageJ software, showed this difference in expression between tumour and control skin to be statistically significant when compared to adjacent normal epidermis (Figure 2D).

### Inherited CYLD‐defective tumours demonstrate a MYB gene expression signature

**Figure 3:**
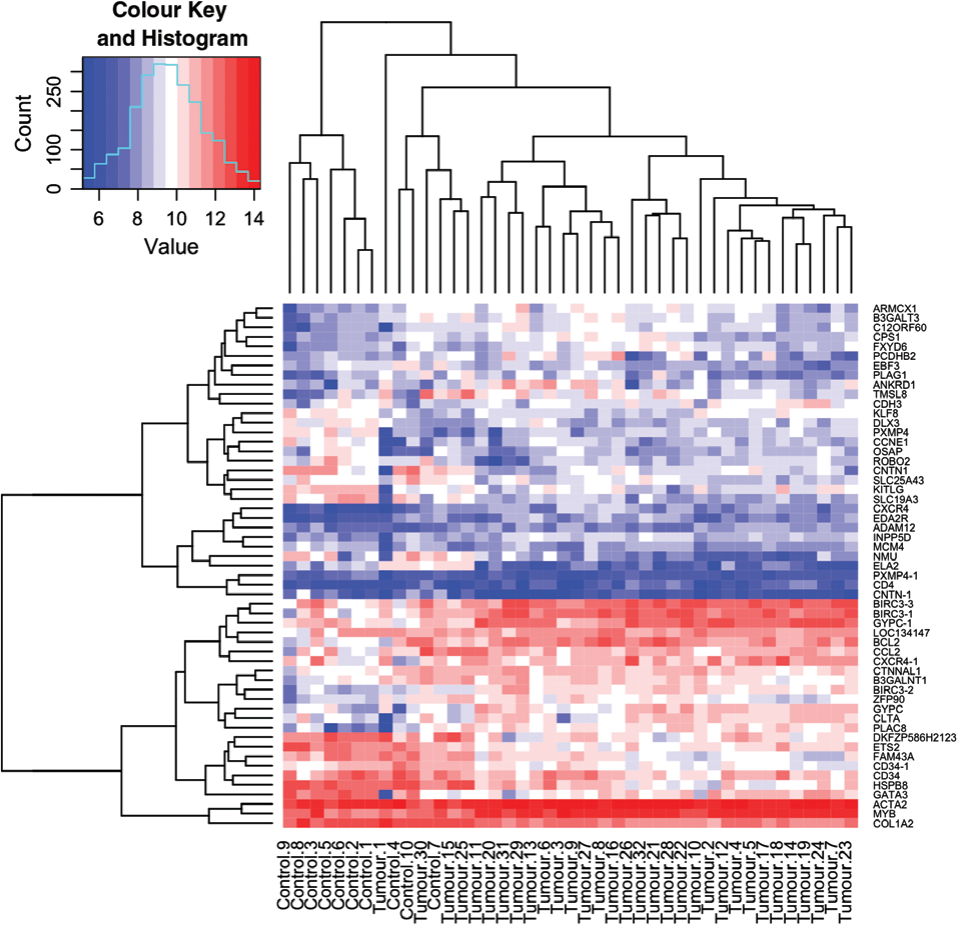
MYB target genes are overexpressed in CYLD‐defective tumours. A heat map plot illustrating the expression levels of MYB and its known target genes in 32 CYLD‐defective tumours and 10 controls. MYB target genes and signalling pathway members that were differentially expressed (p < 0.05) across an average of 32 tumours compared to an average of 10 controls (tumours and controls are indicated at the bottom of the heat map) were included, and transcript expression levels at a single sample level are illustrated in the heat map; red, overexpressed transcripts; blue, transcripts that were under‐expressed; gene names indicated on the right‐hand side of the figure; where data from multiple transcripts for a single gene were available, these are indicated with a numerical suffix (n = 8). Clustering by similarity is shown, as determined by Euclidean distance

We explored the expression of *MYB* and selected MYB target genes following their curation from the published literature and previous experimental data from 32 *CYLD*‐defective tumours 7, in a dataset derived from microarray gene expression studies (no. of transcripts in this set = 263). Genes that were differentially expressed in the pooled signature of 32 *CYLD*‐defective tumours compared to 10 control skin samples, with a threshold for statistical significance of *p <* 0.05 after correction for multiple hypothesis testing, were included (*n =* 48). To visualize the expression of each of these genes at an individual sample level, normalized signal values were plotted on a heat map, following logarithmic transformation (Figure 3). The tumours highlighted the overexpression of *MYB* (fold‐change increase = ×2.04), *BCL2* (fold‐change increase = ×2.34) and *BIRC3* (fold‐change increase = ×3.93) (Table 2). Notably, siRNA knock‐down of MYB in cylindroma cells resulted in down‐regulation of both *BCL2* and *BIRC3* mRNA levels (see supplementary material, Figure S3). Some genes, such as *CD34*, that have been described as MYB target genes in ACC 4 were, however, found to be underexpressed in cylindroma (fold change decrease = ×0.47), whilst genes such as *MAD1L1* were not differentially expressed between tumours and controls (data not shown). Clustering by Euclidean distance separated the majority of cylindromas and spiradenomas (*n =* 30) from the control tissue, apart from two trichoepitheliomas (tumours 1 and 30).

**Table 2:**
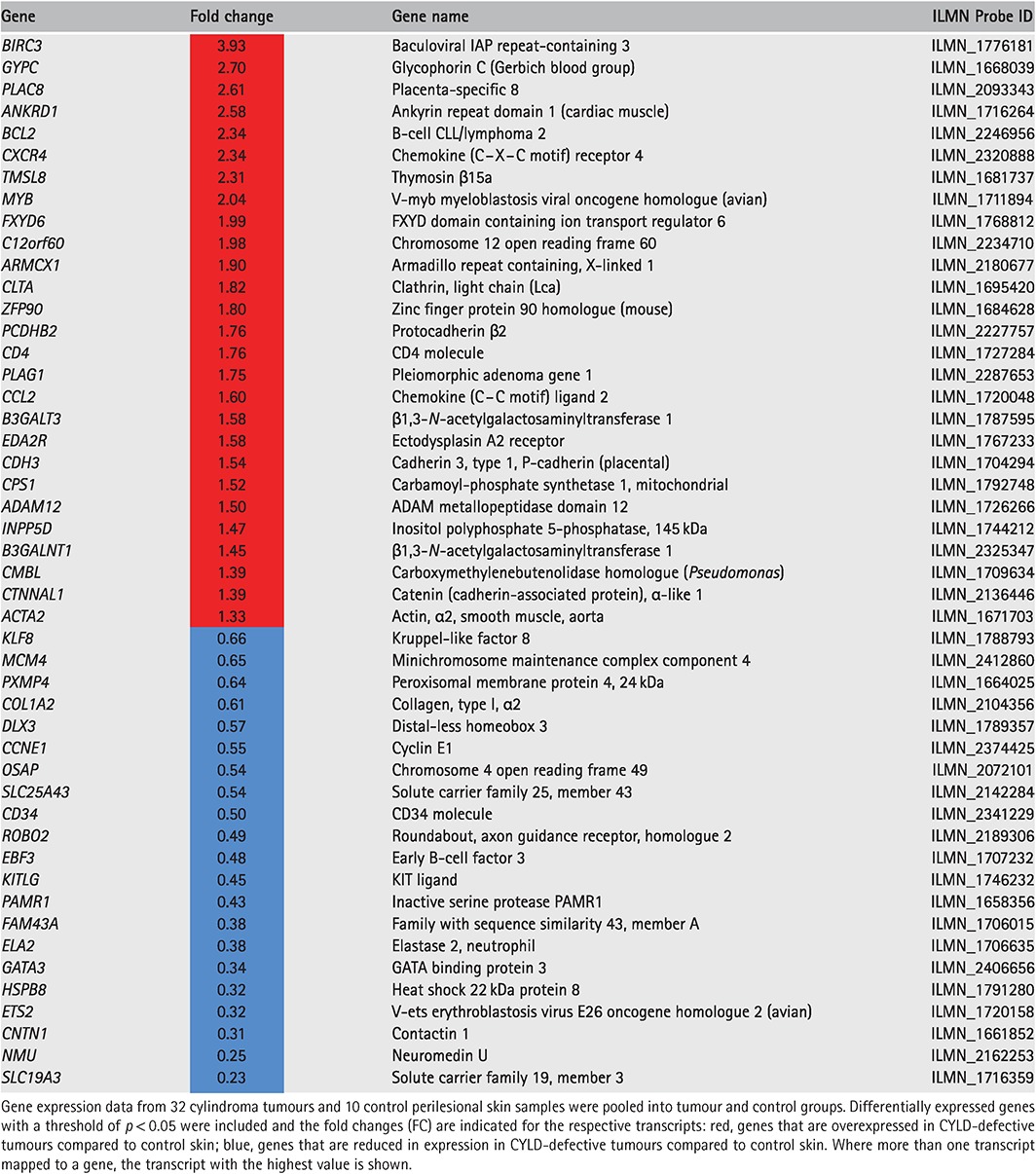
MYB target genes are up‐regulated in CYLD‐defective tumours

### MYB promotes the proliferation of CYLD‐defective cylindroma cells

**Figure 4:**
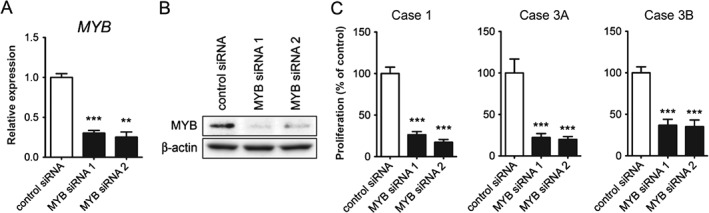
MYB promotes cell proliferation of CYLD‐defective cylindroma cells. (A) RT–qPCR analysis of MYB mRNA expression in cultured cells from case 1, following 48 h of MYB siRNA treatment; ^**^ p < 0.01, ^***^ p < 0.001. (B) Western blot analysis of MYB protein expression in case 1 after 72 h of MYB siRNA treatment. (C) Effects of MYB siRNA treatment (6 days) on the proliferation of cultured, primary cylindroma cells from three individual tumours (cases 1, 3A and 3B); ^***^ p < 0.001. Representative data are shown from one of three independent experiments

To investigate the biological significance of MYB overexpression in cylindroma, given the lack of *MYB–NFIB* fusion transcripts, we used siRNAs to knock down *MYB* mRNA expression in cultured primary *CYLD*‐defective cylindroma cells. Cells from three different primary tumours from two patients undergoing surgical excision, who carried germline mutations in *CYLD* (c.2460delc), were used. Knock‐down of *MYB* mRNA and protein levels (Figure 4A, B) led to a significant decrease in cell proliferation of cylindroma cells in three independent experiments (Figure 4C). These results provide further evidence that MYB is involved in the regulation of cell proliferation of *CYLD*‐defective cylindromas.

## Discussion

We recently reported the presence of *MYB–NFIB* fusion transcripts in sporadic dermal cylindromas 6. Whilst 55% of the tumours were fusion‐positive, some tumours (12%) demonstrated *MYB* activation despite the lack of fusion transcripts. These results suggest that *MYB* activation can replace *MYB–NFIB* fusions in the pathogenesis of a subset of sporadic dermal cylindromas. Similar observations have been made in ACC, where the majority of tumours are *MYB–NFIB*‐positive and a smaller proportion of tumours have *MYB* activation by other mechanisms, such as gene duplication 2, 14. Here, we demonstrate that *MYB* activation is also frequently seen in inherited cylindromas, but strikingly there was a lack of *MYB–NFIB* fusion transcripts in the 23 tumour samples studied. Similarly, we did not find any evidence of rearrangements or gain/amplification of the *MYB* locus in the 10 tumours studied. The finding of *MYB* activation in these inherited tumours is intriguing, since the underlying mechanism behind formation of inherited cylindromas has been shown to be largely limited to a germline mutation of *CYLD* and loss of heterozygosity at the *CYLD* locus. This results in increased NF‐κB signalling, as CYLD is involved in the negative regulation of this oncogenic signalling pathway at several steps, including TRAF2, TRAF6, TAK1 and BCL3 15. Since MYB is a recognised NF‐κB target gene 16 and has NF‐κB binding sites within its promoter (http://bioinfo.lifl.fr/NF‐KB/), it is tempting to speculate that MYB might be activated in a subset of inherited cylindromas as a consequence of aberrant NF‐κB signalling, due to loss of *CYLD* function. In line with this reasoning, it will be interesting to study whether NF‐κB inhibitors can down‐regulate MYB and inhibit the proliferation of *CYLD*‐defective cylindroma cells. Preliminary work with primary cultures of inherited cylindromas demonstrate variable responses, but no down‐regulation of MYB after NF‐κB inhibition, suggesting that a variety of mechanisms may be at play (Rajan *et al.* unpublished data). However, in *MYB–NFIB*‐positive ACC cells, NF‐κB inhibitors have no effect on *MYB* activation (Andersson *et al.*, unpublished data).

The occurrence of *MYB–NFIB* fusion transcripts in sporadic cylindromas and their absence in inherited cases may reflect the timing of a gained *MYB* activation state. In patients with germline mutations in *CYLD*, the typical age of presentation of the first tumour is 16 years 17. In these patients, loss of heterozygosity (LOH) at the *CYLD* locus is seen in 76% of tumours 18, and some of the remaining cases have compound heterozygous changes affecting *CYLD* 5. By contrast, sporadic cylindromas typically present beyond the fifth decade of life. STR studies have highlighted that approximately 50% of these tumours (*n =* 14) demonstrate LOH at 16q, the locus of *CYLD* 18, making the recent finding that 55% of tumours in a similar sized study (*n =* 12) demonstrate *MYB–NFIB* fusions of interest 6. There are limited data on the sequencing of coding exons of sporadic cylindromas, but some have indeed been shown to carry mutations in *CYLD* 5. However, it remains to be determined whether *MYB–NFIB* fusion‐positive sporadic dermal cylindromas also carry bi‐allelic *CYLD* mutations, as these two changes could potentially act synergistically in promoting cylindroma development.

To further examine the role of MYB overexpression in the molecular pathogenesis of inherited cylindromas, we silenced the expression of MYB in *CYLD*‐defective cylindroma cells, using RNA interference. This resulted in a significant inhibition of MYB target genes and proliferation of cylindroma cells from three independent tumours, suggesting that *MYB* activation drives the growth of *CYLD*‐defective cylindromas and potentially also the growth of *MYB–NFIB*‐positive sporadic cylindromas. This is in line with previous studies of *Cyld^−/−^* mice, which do not spontaneously develop tumours but are highly susceptible to chemically induced skin tumours 19, indicating that additional molecular events besides *Cyld* inactivation are required for tumour formation.

Interestingly, parallels between inherited cylindromas and ACC may extend beyond MYB, as was recently highlighted with the demonstration that both tumour types overexpress TRKC 7, 20. *CYLD*‐mutation carriers also develop salivary gland tumours, and there are reports of areas of ACC‐like differentiation arising within cylindromas 21. Furthermore, recent studies of the mutational landscape in ACC have shown that *CYLD* is mutated in 4% of these tumours 22. It is also intriguing that *MYB–NFIB* fusions are seen in ACCs of the breast and salivary glands as well as in sporadic cylindromas, suggesting that *MYB* activation may be central to the histological patterning of these tumours. Our data highlight the potential cross‐cutting insights that can be obtained from the study of these rare tumours. MYB is an attractive therapeutic target in ACC and treatments that are developed for ACC may hence be of relevance for patients with both sporadic and inherited cylindromas, and vice versa.

## Author contributions

NR and GS conceived the study; NR, NS, KH, TV, MA and AF carried out experiments; and NR, CJL, AA, DK, MA and GS conceived experiments and analysed data. All authors were involved in writing the paper and had final approval of the submitted and published versions.

**Supplementary material on the internet**

The following supplementary material may be found in the online version of this article:

**Figure S1.** MYB protein expression in inherited CYLD‐defective and sporadic tumours

**Figure S2.** MYB protein expression in skin

**Figure S3.** Effects of *MYB* siRNA knockdown on MYB targets

**Table S1.** Primer sequences of PCR‐primers used to detect *MYB–NFIB* fusion transcripts

## Supporting information

MYB protein expression in inherited CYLD‐defective and sporadic tumours. FFPE tissue sections of cylindromas and spiradenoma and perilesional control skin were immunostained with an anti‐MYB antibody. (A, B) MYB protein expression in (A) inherited cylindroma and (B) inherited spiradenoma, demonstrating nuclear localization and increased intensity of staining when compared to perilesional tissue. (C, D) MYB protein expression in (C) sporadic cylindromas that are MYB–NFIB fusion‐positive and (D) MYB–NFIB fusion‐negative show similar patterns of MYB expression and localization (cases 2 and 9 in [6])

MYB protein expression in skin. (A) perilesional control skin from a CYLD mutation carrier and (B) age‐matched unaffected control demonstrates cytoplasmic localization in the majority of cells

Effects of MYB siRNA knockdown on MYB targets. BCL2 and BIRC3 are down‐regulated following MYB knockdown in primary cultured CYLD‐defective cylindroma cells (case 1)

Primer sequences of PCR primers used to detect MYB–NFIB fusion transcripts
