# Supplementary figures and images for: Overexpression of MYB drives proliferation of CYLD‐defective cylindroma cells
Source: J Pathol. 2016 Apr 21;239(2):197–205. doi: 10.1002/path.4717 (PMC4869681; doi:10.1002/path.4717)

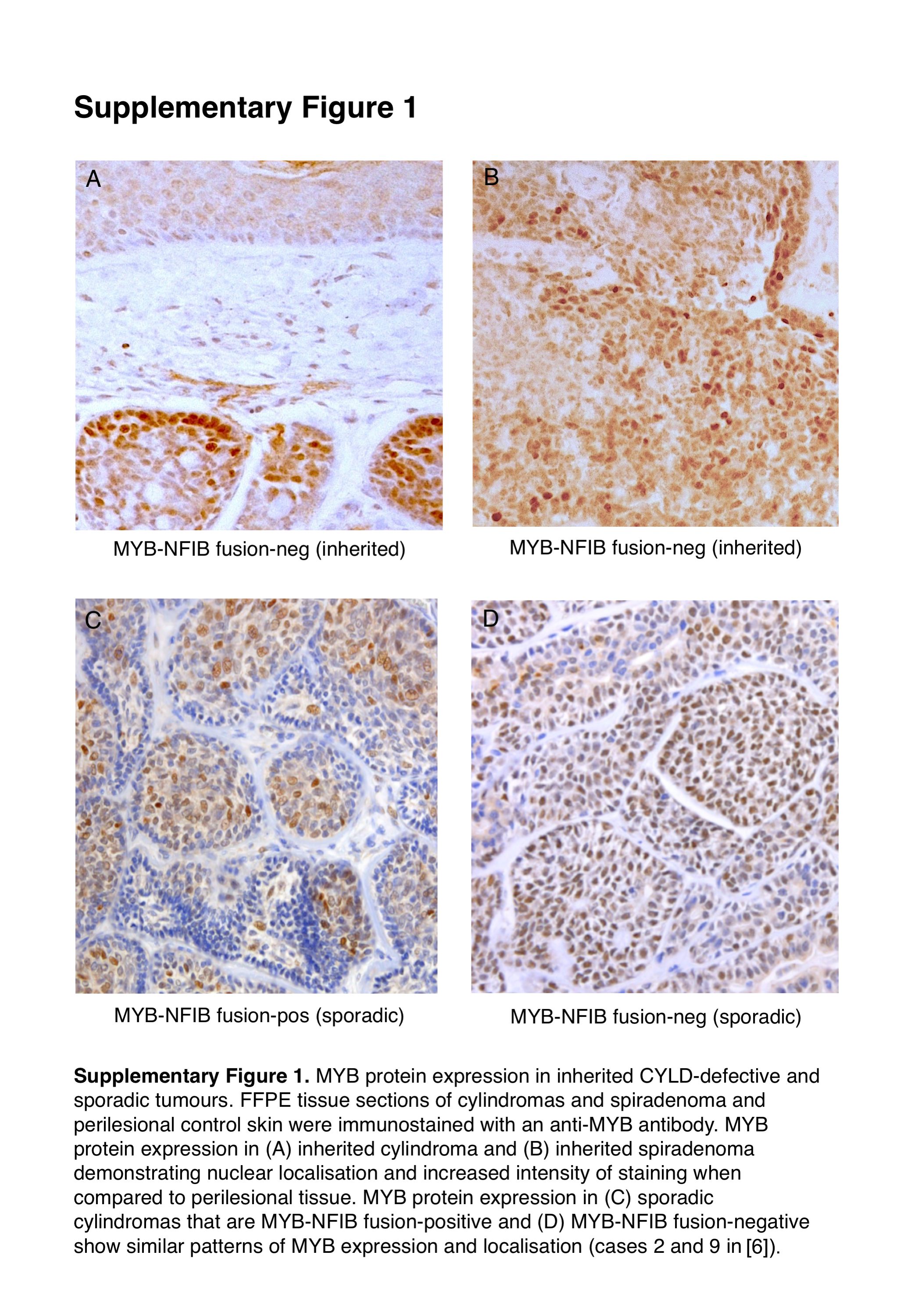

Supplement: Supplementary file 1 — MYB protein expression in inherited CYLD‐defective and sporadic tumours. FFPE tissue sections of cylindromas and spiradenoma and perilesional control skin were immunostained with an anti‐MYB antibody. (A, B) MYB protein expression in (A) inherited cylindroma and (B) inherited spiradenoma, demonstrating nuclear localization and increased intensity of staining when compared to perilesional tissue. (C, D) MYB protein expression in (C) sporadic cylindromas that are MYB–NFIB fusion‐positive and (D) MYB–NFIB fusion‐negative show similar patterns of MYB expression and localization (cases 2 and 9 in [6]) [file PATH-239-197-s004.tif]

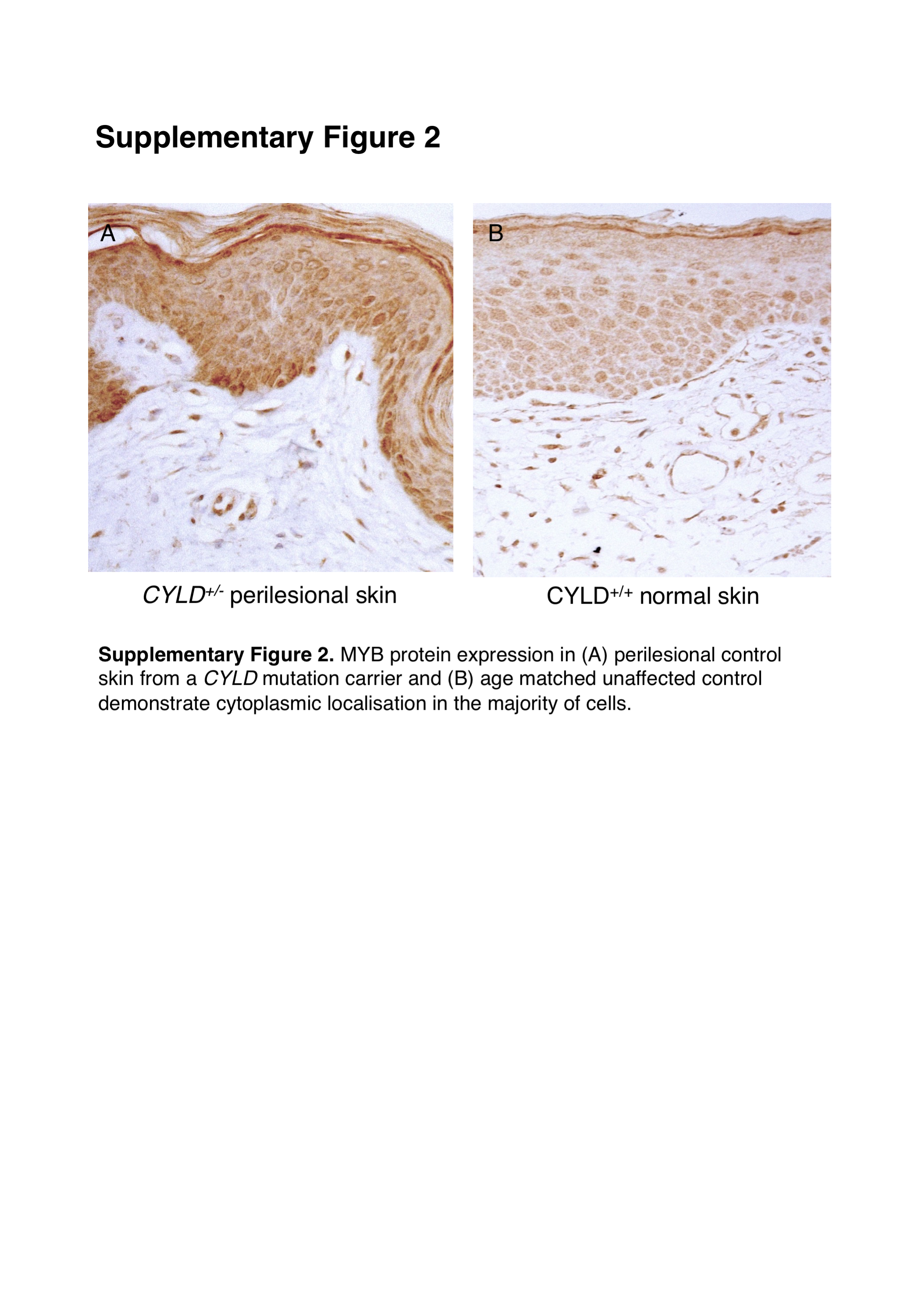

Supplement: Supplementary file 2 — MYB protein expression in skin. (A) perilesional control skin from a CYLD mutation carrier and (B) age‐matched unaffected control demonstrates cytoplasmic localization in the majority of cells [file PATH-239-197-s002.tiff]

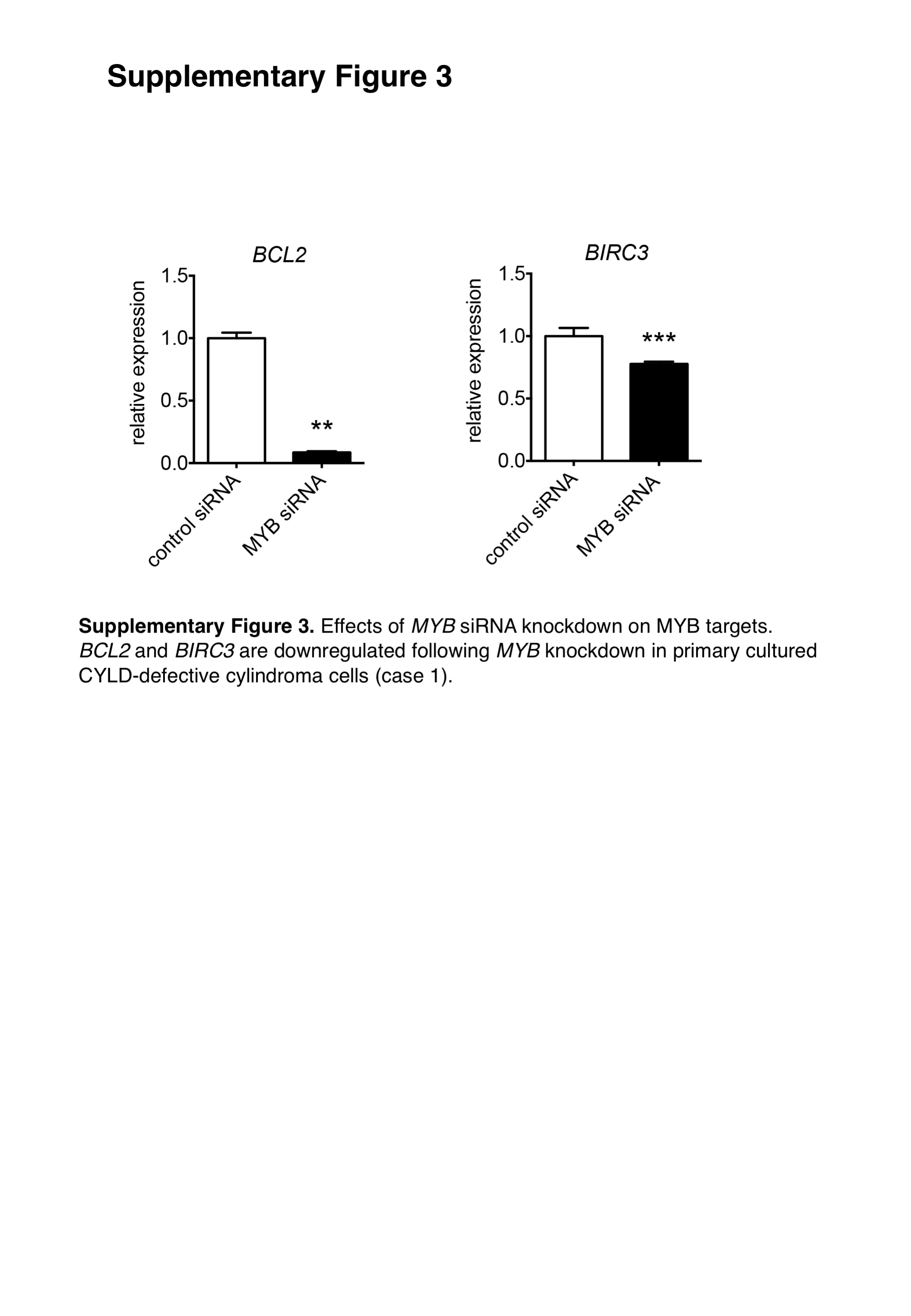

Supplement: Supplementary file 3 — Effects of MYB siRNA knockdown on MYB targets. BCL2 and BIRC3 are down‐regulated following MYB knockdown in primary cultured CYLD‐defective cylindroma cells (case 1) [file PATH-239-197-s003.tiff]
