# Supplementary material for: Overexpression of MYB drives proliferation of CYLD‐defective cylindroma cells
Source: J Pathol. 2016 Apr 21;239(2):197–205. doi: 10.1002/path.4717 (PMC4869681; doi:10.1002/path.4717)
Supplement: Supplementary file 4 — Primer sequences of PCR primers used to detect MYB–NFIB fusion transcripts [file PATH-239-197-s001.doc]

**Table S1.** Primer sequences of PCR primers used to detect *MYB–NFIB* fusion transcripts

| **No.** | **Marker** | **Forward** | **Reverse** | **Accession No.** |
| --- | --- | --- | --- | --- |
| 363 | MYBex5-697F | GGCAGAAATCGCAAAGCTAC |  | NM_005375 |
| 365 | MYBex9-1282F | TGGCTCCCTACCTGAAGAAA |  | NM_005375 |
| 366 | MYBex9-1334F | CAGGGCACCATTCTGGATAA |  | NM_005375 |
| 367 | MYBex11-1554F | CCCCAGCTATCAAAAGGTCA |  | NM_005375 |
| 368 | MYBex11-1569F | GGTCAATCTTAGAAAGCTCTC |  | NM_005375 |
| 369 | MYBex12-1693F | GCAGGATGTGATCAAACAGG |  | NM_005375 |
| 370 | MYBex14-1925F | GCACCAGCATCAGAAGATGA |  | NM_005375 |
| 381 | NFIBex9-1952R |  | GTGCTGCAATTGCTGGTCTA | NM_005596 |
| 382 | NFIBex9-2078R |  | CTATTTCCCAGCGGACTTCA | NM_005596 |
| 391 | MYBex10-1432F | CTCAGACTTGGAAATGCCTTC |  | NM_005375 |
| 495 | NFIBex8c-1067R |  | GGTCCAGTCACAAATCCTCAG | BX648416 |
| 496 | NFIBex8c-1096R |  | GGGTATAAATGCCTGCCGTT | BX648416 |
